# Neuropsychiatric implications of covid-19 infection: A case report

**DOI:** 10.1192/j.eurpsy.2022.1317

**Published:** 2022-09-01

**Authors:** P. Coucheiro Limeres, A. Franco Soler, A. Cerame, S. Maldonado Orellana

**Affiliations:** Hospital Universitario José Germain, Psychiatry Department, Leganés, Spain

**Keywords:** NEUROPSYCHIATRIC IMPLICATIONS, COVID19, Seizures

## Abstract

**Introduction:**

During the COVID19 pandemic numerous cases of neuropsychiatric complications were reported as a result of COVID19.

**Objectives:**

Presentation of a clinical case and literature review of new cases of neuropsychiatric complications after SARS-CoV2.

**Methods:**

We present the case of a 43-year-old woman in follow-up for 15 years borderline personality disorder who was diagnosed with SARS-CoV2 pneumonia without signs of severity. Throughout the admission the patient, who had no history of epilepsy or other neurological affections, presented up to 5 generalized tonic-clonic seizures during 15 days.

**Results:**

In the ECG was evidenced intercritical epileptiform activity predominantly right frontotemporal. No analytical alterations were observed, neither in the imaging tests (cranial MRI and CT). Lumbar puncture was normal. During the admission, he presents an affective deterioration, with generalized impoverishment, decreased functional autonomy and hearing voices without structured delusional ideation. No previous psychotic history. Her previous treatment with Sertraline 100mg was suspended and valproic acid was added (1300 mg/day) which, being insufficient in the control of seizures, was necessary to boost with Levetiracetam (1000 mg/12h). Risperidone 3 mg and Diazepam 5 mg/8h were added to control psychotic symptoms. In the subsequent follow-up, previous antipsychotic treatment was gradually discontinued. The patient evolved favorably without new psychotic symptoms and clinical stability was observed with euthymia.

**Conclusions:**

Taking care of these complications it is necessary to avoid misdiagnosing. It is essential to expand the study of this entities in the context of COVID19 in order to increase knowledge and to be able to carry out an adequate approach and follow-up.

**Disclosure:**

No significant relationships.

